# Supplementary material for: Deterministic transfer of optical-quality carbon nanotubes for atomically defined technology
Source: Nat Commun. 2021 May 25;12:3138. doi: 10.1038/s41467-021-23413-4 (PMC8149403; doi:10.1038/s41467-021-23413-4)
Supplement: Supplementary file 1 — Supplementary Information [file 41467_2021_23413_MOESM1_ESM.pdf]

Supplementary Information

Deterministic transfer of optical-quality carbon nanotubes for atomically defined technology

Keigo Otsuka[1†*], Nan Fang[1], Daiki Yamashita[2], Takashi Taniguchi[3], Kenji Watanabe[4], Yuichiro K. Kato[1,2,*]

[1]Nanoscale Quantum Photonics Laboratory, RIKEN Cluster for Pioneering Research, Saitama 351-0198, Japan

[2]Quantum Optoelectronics Research Team, RIKEN Center for Advanced Photonics, Saitama 351-0198, Japan

[3]International Center for Materials Nanoarchitectonics, National Institute for Materials Science, Ibaraki 305-0044, Japan

[4]Research Center for Functional Materials, National Institute for Materials Science, Ibaraki 305-0044, Japan

†Present address: Department of Mechanical Engineering, The University of Tokyo, Tokyo 113-8656, Japan

---

* email: otsuka@photon.t.u-tokyo.ac.jp, yuichiro.kato@riken.jp

Supplementary Note 1:

Photoluminescence measurements during nanotube transfer

We use a homebuilt micro-photoluminescence (PL) measurement system to characterize carbon nanotubes (CNTs). To facilitate the transfer process, reflectivity of a laser beam and a coaxial wide-field LED illumination, as well as PL from CNTs are simultaneously monitored in real time. For example, the laser reflectivity provides the precise position of the receiving substrate in three coordinates. When using a transparent stamp, the reflectivity shows an interference pattern while approaching the substrate (Supplementary Fig. 1a). PL is monitored to non-destructively locate CNTs, identify the chirality, and capture changes in the surrounding environment of the CNTs. Note that we use a closed-loop translation stage to move the substrate, but an open-loop stage for the stamp. The reproducibility of the open-loop stage predominantly limits the position accuracy of the transfer process.

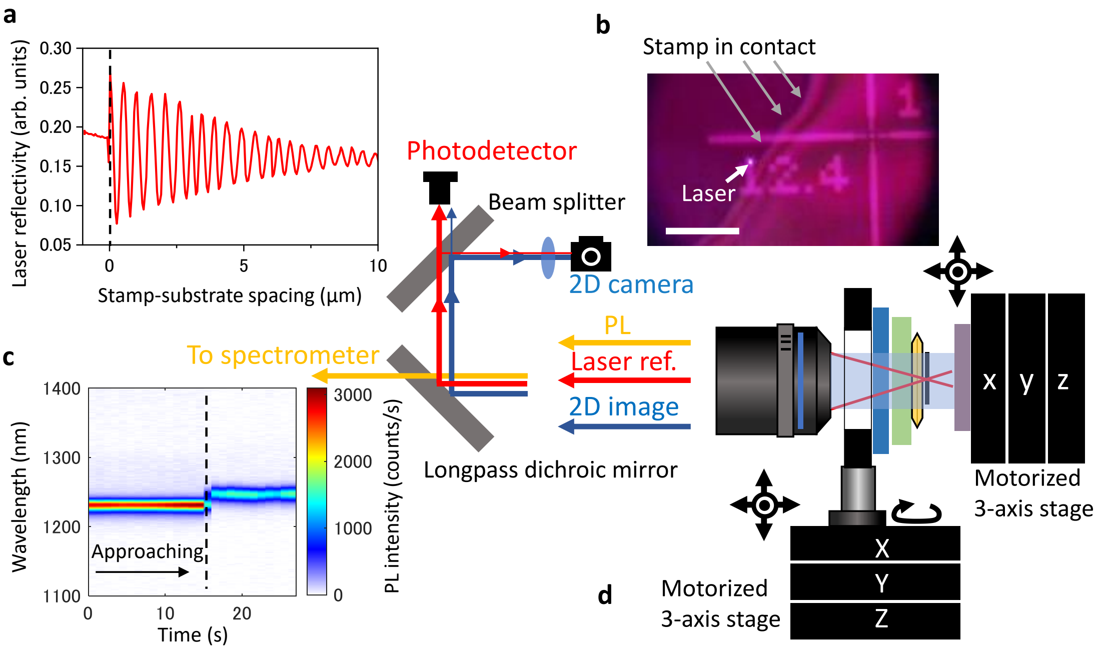

Supplementary Fig. 1 | Overview of the anthracene-assisted transfer system incorporated in a micro-PL setup. a, Reflectivity of the laser beam as a function of the spacing between a stamp and a substrate. As the stamp approaches, the amplitude of interference increases and becomes stable after the contact. **b**, Wide-field optical image of the substrate surface. The left half is in contact with the stamp. Scale bar is 20 µm. **c**, Transition of PL spectra of an air-suspended CNT while approaching an anthracene crystal to the CNT. Dashed line represents the contact of the CNT and the anthracene crystal, which induces a redshift of the emission spectra. **d**, Schematic of the transfer system in the optical setup that allows for in-situ confocal PL measurements and reflection imaging.

Supplementary Note 2:

Crystal growth of anthracene by in-air sublimation

We employ a simple crystal growth technique, in which the temperature at a growth substate is controlled by the distance to a heated substrate with molecular powder sources [1]. The crystals are grown through sublimation in air. We find that a coating of glass slides with alcohol-based marker ink (KOKUYO, PM-41B) suppresses the nucleation of anthracene crystals (blue in Supplementary Fig. 2a), which leads to the growth of large-area and thin crystals. Although the information on the dye (ink) is unavailable, the dye molecules are likely to have a large interfacial free energy with the (001) plane of the crystals, which is in contact with CNTs during the transfer.

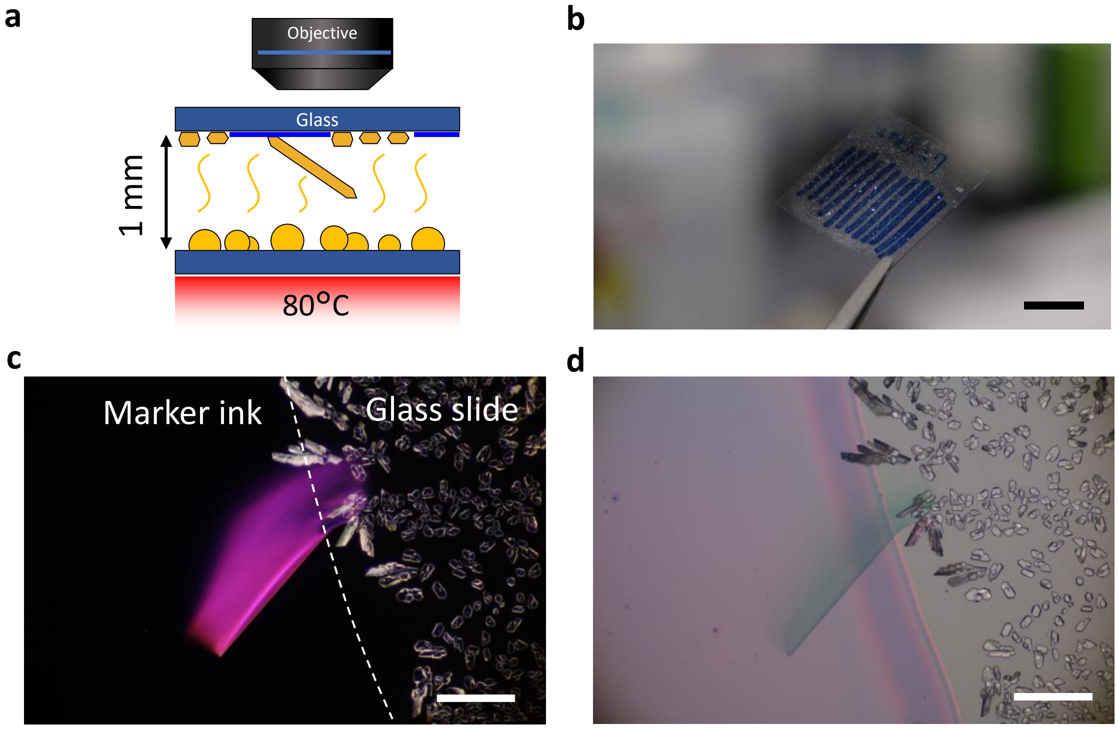

Supplementary Fig. 2 | Anthracene crystal growth. a, Schematic for crystal growth of anthracene in air. **b**, Photograph showing a glass slide after the crystal growth. Blue lines are drawn with a commercially available marker, on which the nucleation of crystals is suppressed. Scale bar is 10 mm. **c**,**d**, Optical micrographs of anthracene crystals under dark-field (**c**) and bright-field (**d**) illumination. Scale bars are 100 μm.

Supplementary Note 3:

Diameter dependence of the transfer efficiency

Unlike the polymer-mediated transfer that involves wet etching of the growth substrates [2], the overall transfer efficiency of the dry stamping method is far less than 100%. Interestingly, chirality distribution is different for the as-grown suspended CNTs and the transferred CNTs (Supplementary Fig. 3a). Note that the transferred CNTs are grown on a flat SiO_2/Si surface from unpatterned iron catalyst. By comparing the chirality distribution between the as-grown and transferred samples (Supplementary Fig. 3b), we find a strong diameter dependence in the transfer efficiency (Supplementary Fig. 3c). As the transfer efficiency for the thinnest CNTs is obviously <100%, the efficiency for the CNTs with the diameter of ~1.2 nm or more should be <1%. Considering the crystalline nature of the anthracene stamp, there also could be a chiral angle dependence, as well as a dependence on tube-crystal orientation matching. The current results show a slight dependence on these parameters, but further study is needed.

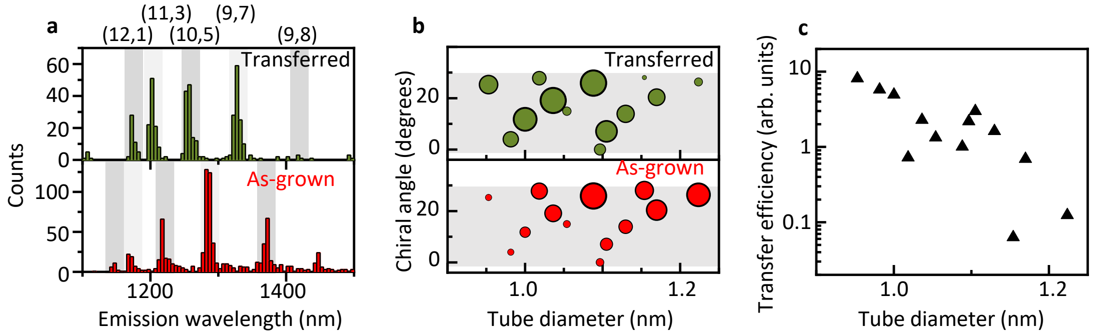

Supplementary Fig. 3 | Chirality dependence of CNTs transferred via anthracene crystals. a, Histograms of emission wavelengths for the CNTs on PMMA or suspended over trenches excited at 790 (top) and 780 nm (bottom), respectively. **b**, Chirality distribution as a function of tube diameter and chiral angle for transferred (top) and as-grown (bottom) CNTs. The area of the circles represents the population. In total, the chiral indices of 771 and 1307 tubes are assigned for the transferred and as-grown samples, respectively. **c**, Transfer efficiency *versus* tube diameter, where ratios of the abundance of transferred CNTs to as-grown CNTs in **b** are plotted.

Supplementary Note 4:

Length of transferred CNTs on polymer

The transferred CNTs shown in Fig. 3–5 and Supplementary Fig. 3 are grown simultaneously with the as-grown suspended ones using similar catalyst (evaporated iron). The length of those CNTs should therefore be more or less the same. In SEM and PL images in Supplementary Fig. 4, the typical length of the transferred CNTs is 1–5 µm and similar to those suspended over trenches. We also did not find excessive bundle formation of the transferred CNTs, indicating that the PL intensity comparison between the as-grown suspended CNTs and the transferred CNTs is not biased much by the tube length difference.

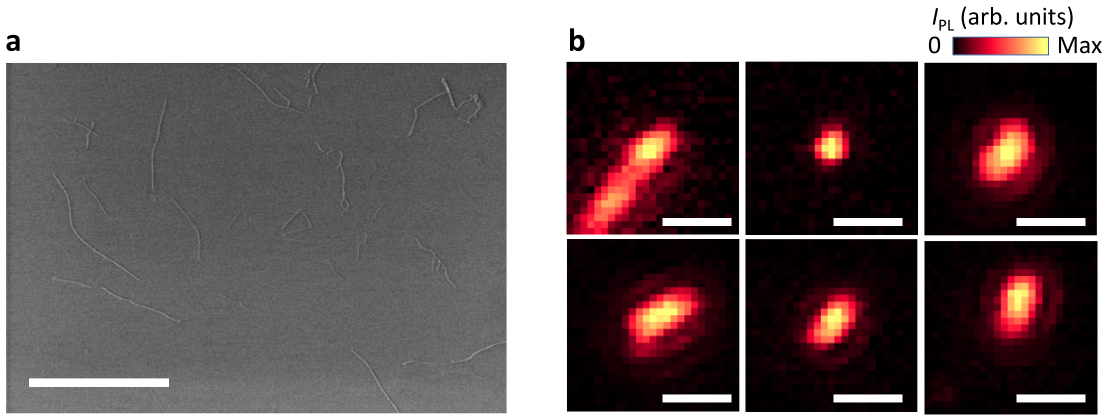

Supplementary Fig. 4 | Images of transferred CNTs on polymer. a, SEM image of transferred CNTs on PMMA. CNTs are originally grown on flat a SiO_2/Si substrate and randomly orientated. **b**, Typical PL images of the CNTs. Scale bars in **a** and **b** are 5 and 2 µm, respectively.

Supplementary Note 5:

Changes to PL spectra before and after anthracene contact

We have checked that PL of CNTs is not significantly quenched by the contact with anthracene crystals. Supplementary Fig. 5 shows PLE maps of the same air-suspended CNT before and after the anthracene contact, which were measured through a glass/PDMS/anthracene stamp. The PL trace of this CNT during the contact formation is found in Supplementary Fig. 1c. The contact with anthracene induced E_{11} and E_{22} redshifts as well as a slight spectral broadening. The integrated PL intensity at the resonance is 2.01×10^4 and 1.83×10^4 counts/s before and after the contact, respectively. Although the collection efficiency of nanotube PL can be changed by placing materials with a high refractive index on top of the CNTs, the reduction of the PL intensity is insignificant for performing deterministic transfer of CNTs on anthracene under *in-situ* PL monitoring.

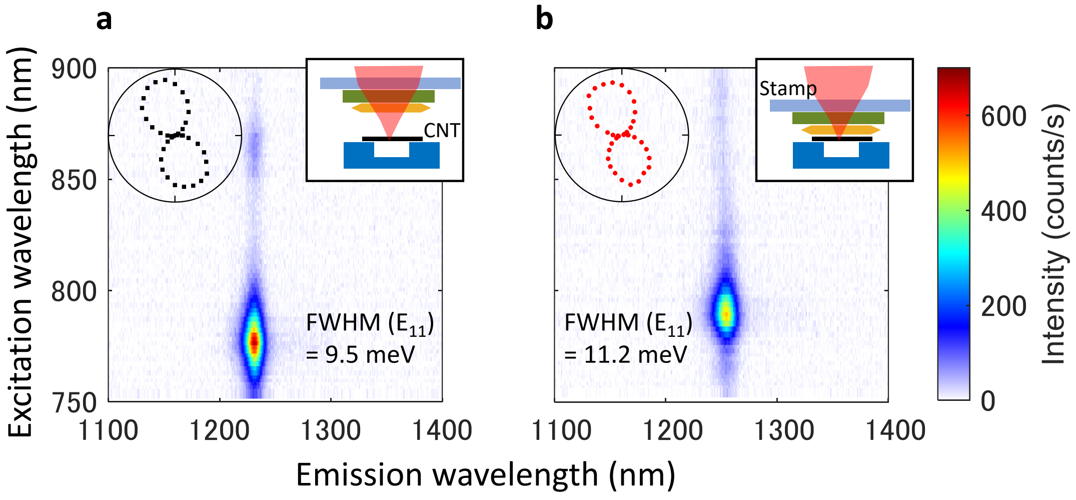

Supplementary Fig. 5 | Spectral tracing of a CNT. a,**b**, PLE maps of the same CNT before (**a**) and after (**b**) the contact with an anthracene crystal. Inset: Polarization angle-dependent PL intensity. The configuration of the sample and measurement is also shown at the top right. *P* = 10 μW.

Supplementary Note 6:

Statistics of PL properties for various CNT samples

In addition to Fig. 3a, we have performed statistical PL measurements for two more samples: CNTs on anthracene and on SiO_2/Si with P = 100 μW. In Supplementary Fig. 6a, histograms of $\log_{10}(I_{PL})$ are shown for each sample. Note that the excitation power here is much larger than that used in Fig. 3a (P = 5 μW), and PL enhancement factors are smaller due to efficient exciton-exciton annihilation in high PL efficiency samples. The detector noise is centered at 0 counts/s and follows a normal distribution in linear scale, but in log scale, $\log_{10}(I_{PL})$ of the noise peaks at ~3 due to uneven binning width. Similarity of the frequency corresponding to noise indicates that the density of CNTs in each sample are comparable. I_{PL} of all the samples above the noise level is well fitted with a log-normal distribution (solid lines). I_{PL}, at which the relative frequency is 10^{-3} for each sample, is indicated by the vertical lines in Supplementary Fig. 6a. The PL intensity of the CNTs on anthracene is close to that on polystyrene, while that on SiO_2/Si lies between the as-grown CNTs on quartz and the transferred CNTs on PMMA. Consistent with the previous study [3], the removal of the strong interaction with growth substrates suppresses exciton quenching, but polar substrates have stronger quenching effect than polymers.

Spectral linewidths of each sample follow a normal distribution (Supplementary Fig. 6b). When we fit the emission spectra of the three types of CNTs with a Voigt function, Lorentzian (related to exciton dephasing) and Gaussian components (related to inhomogeneity) can be obtained (Supplementary Fig. 6c,d). Because no correlation is found for the PL intensity and the linewidth among CNTs in each sample (Supplementary Fig. 6e), the variety in the linewidth may originate from environmental inhomogeneity within each sample rather than shortened dephasing time caused by quenching sites. We speculate that substrate flatness may influence the environmental inhomogeneity. Compared with the surface of PMMA films, that of anthracene is flat because of the single crystalline nature, whereas air-suspended CNTs are free from such substrate effects.

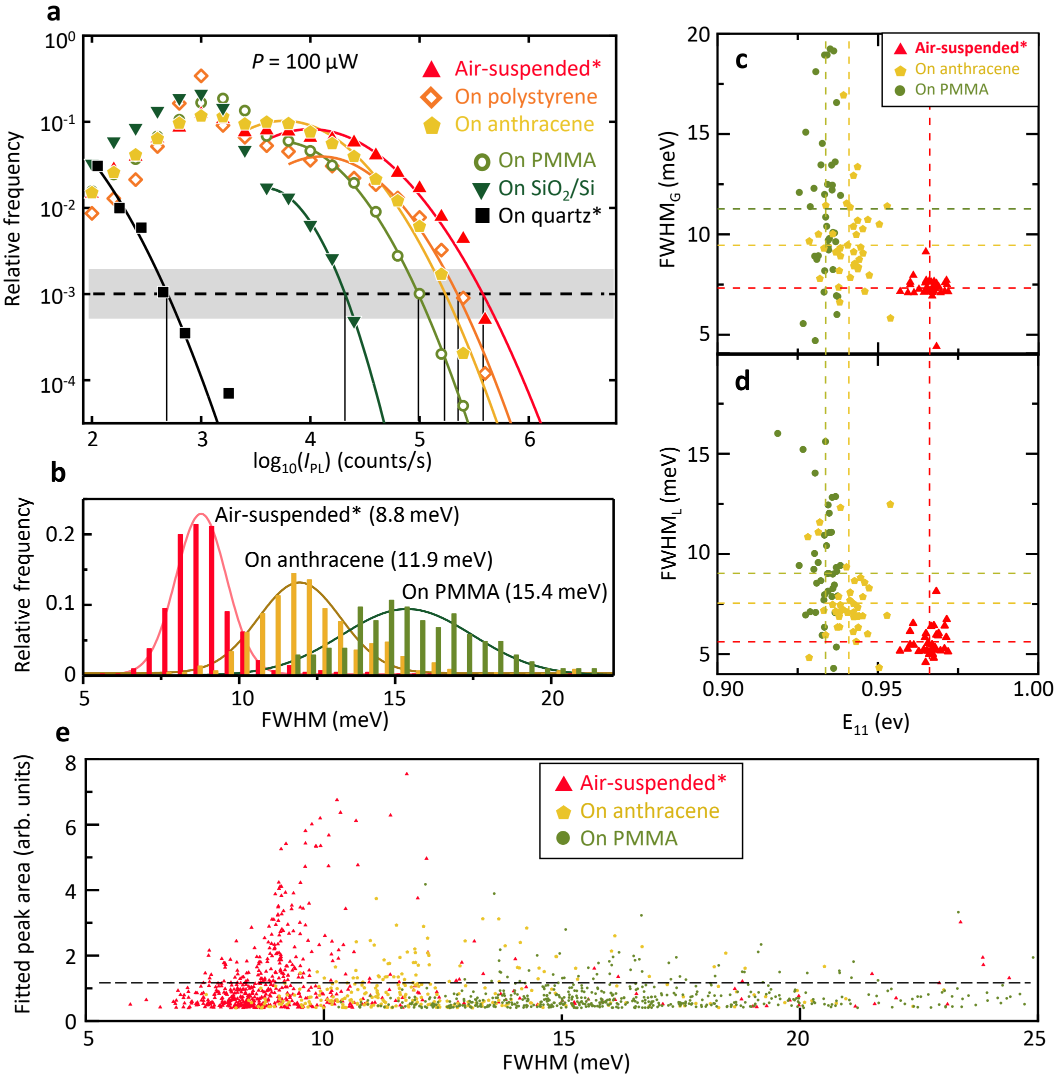

Supplementary Fig. 6 | Statistical study on optical quality of various CNT samples. a, Histograms of $\log_{10}(I_{PL})$ at P = 100 μW for six types of CNTs. Peaks around $\log_{10}(I_{PL})$ = 3 originate from detector noise, and the other regions are fitted by log-normal distributions (solid lines). The frequency range 5×10^{-2} to 2×10^{-3} is shown in gray. **b**, Histograms of PL linewidths for three types of CNTs. Solid lines represent Gaussian fits. The sample name with asterisks represent as-grown CNTs; otherwise, the CNTs are transferred *via* anthracene. **c**,**d**, Full width at half maximum (FWHM) of Gaussian components (**c**) and Lorentzian components (**d**) of the Voigt function fitting for PL spectra of three types of CNTs. Dashed lines represent the averages. **e**, Fitted peak area against the spectral FWHM. Dashed line represents the threshold for data selection.

Supplementary Note 7:

PL intensity widely ranges due to stochastic quenching sites

As shown in Supplementary Fig. 6, the PL intensity of individual CNT follows a log-normal distribution, and ranges over a few orders of magnitude even among as-grown suspended CNTs over the trenches with similar widths. As the chance of quenching site creation during CVD should be equal for all CNTs, the density of quenching sites in a nanotube is expected to follow a normal distribution (or a Poisson distribution). The probability f for having λ quenching defects in a nanotube can be written as

$$f(\lambda) = \frac{1}{\sqrt{2\pi}\sigma}\exp\left(-\frac{(\lambda - N)^2}{2\sigma^2}\right), \quad (1)$$

with N and σ being the average number of quenching sites and its standard deviation, respectively. As shown in Supplementary Fig. 6, when the frequency g of a CNT with I' $\equiv \ln(I_{PL})$ follows

$$g(I') = \frac{1}{\sqrt{2\pi}\alpha\sigma}\exp\left(-\frac{(I' - I'_N)^2}{2\alpha^2\sigma^2}\right) \quad (2)$$

with I'_N being the average of $\ln(I_{PL})$, it implies that I_{PL} of a CNT with λ quenching sites is

$$I_{PL} = I_0 \exp(-\alpha\lambda), \quad (3)$$

where α is a constant and $I_0 = \exp(I'_N + \alpha N)$ represents the PL intensity of defect-free nanotubes.

Assuming stochastic formation of quenching sites with an average number N in a single nanotube that recombine excitons nonradiatively, we have performed Monte Carlo simulation to quantify the effect of the quenching sites on the PL intensity from a CNT (see Refs. [4,5] for details). In the simulations, a nanotube length L = 2 μm, a $1/e^2$ laser beam radius r = 400 nm (FWHM of ~470 nm), and an intrinsic exciton diffusion length l = 700 nm [4] are used, while excitons encountering nanotube edges, quenching sites, and other excitons recombine immediately.

Supplementary Fig. 7a shows simulated PL intensity as a function of λ, and the result is well fitted with Supplementary Equation 3 (red) for $\lambda > 3$ where the length of CNT segments is smaller than exciton diffusion length. Error bars represent the standard deviation, which originates from the random position of quenching sites as shown in Supplementary Fig. 7b. As discussed above, Monte Carlo simulation shows that the frequency of having λ quenching sites follows a normal distribution (Supplementary Fig. 7c), and the histograms of PL intensity with different N can be well fitted with a log-normal distribution (Supplementary Fig. 7d), in good agreement with the experiments. Note that the number of quenching sites λ follows a Poisson distribution which can be approximated well by a normal distribution when the average number is large. Supplementary Equation 1 does not hold for small N, resulting in the deviation from the

fitting lines at large I_{PL} as observed both in the experiments (the as-grown suspended CNTs and the CNTs on polystyrene) and the simulation (N/L = 2 and 3 μm^{-1}). The $\log_{10}(I_{PL})$ of 5.6 (= 4×10^5 counts/s) in Supplementary Fig. 6 is therefore likely obtained from defect-free CNTs. We should also note that the following equation

$$I_{PL} = \sum_{i}^{2} I_i \exp(-\alpha_i \lambda_i) \quad (4)$$

reproduces the result in Supplementary Fig. 7a better, and a histogram of I_{PL} with this equation can be also fitted by a log-normal function over a wide range. Compared with the previously reported effective length dependence of PL quantum yield, where the quantum yield is proportional to the square of effective tube lengths [6,7], Supplementary Equations 3 and 4 fit the simulation result (Supplementary Fig. 7a) better and thereby the experimental I_{PL} distribution (Supplementary Fig. 6a). This is probably because the tube segments separated by quenching sites have uneven lengths due to their random defect formation.

We note that the biexponential dependence in our simulation is more evident in a log-scale (Supplementary Fig. 7e), although the model is rather phenomenological. When the intrinsic exciton diffusion l is short (top), or exciton generation rate $g_0\tau$ is large (bottom; effective diffusion length is short due to exciton-exciton annihilation), the dependence is well reproduced by a single exponential curve. The slopes at small λ/L and large λ/L in Supplementary Fig. 7e are likely to reflect a nanotube-length-limited regime and a quenching-limited regime, respectively, as observed in PL intensity profiles along the tube axis and their FWHM (Supplementary Fig. 7f). In the quenching-limited regime, the PL intensity profile becomes the same as the excitation intensity profile (that is, Gaussian function in the simulations). We should stress that the experimental P = 5 μW roughly corresponds to $g_0\tau$ = 3 [5], and the measurements in Supplementary Fig. 6a (measured at P = 100 μW) should follow the single exponential model in Supplementary Equation 3.

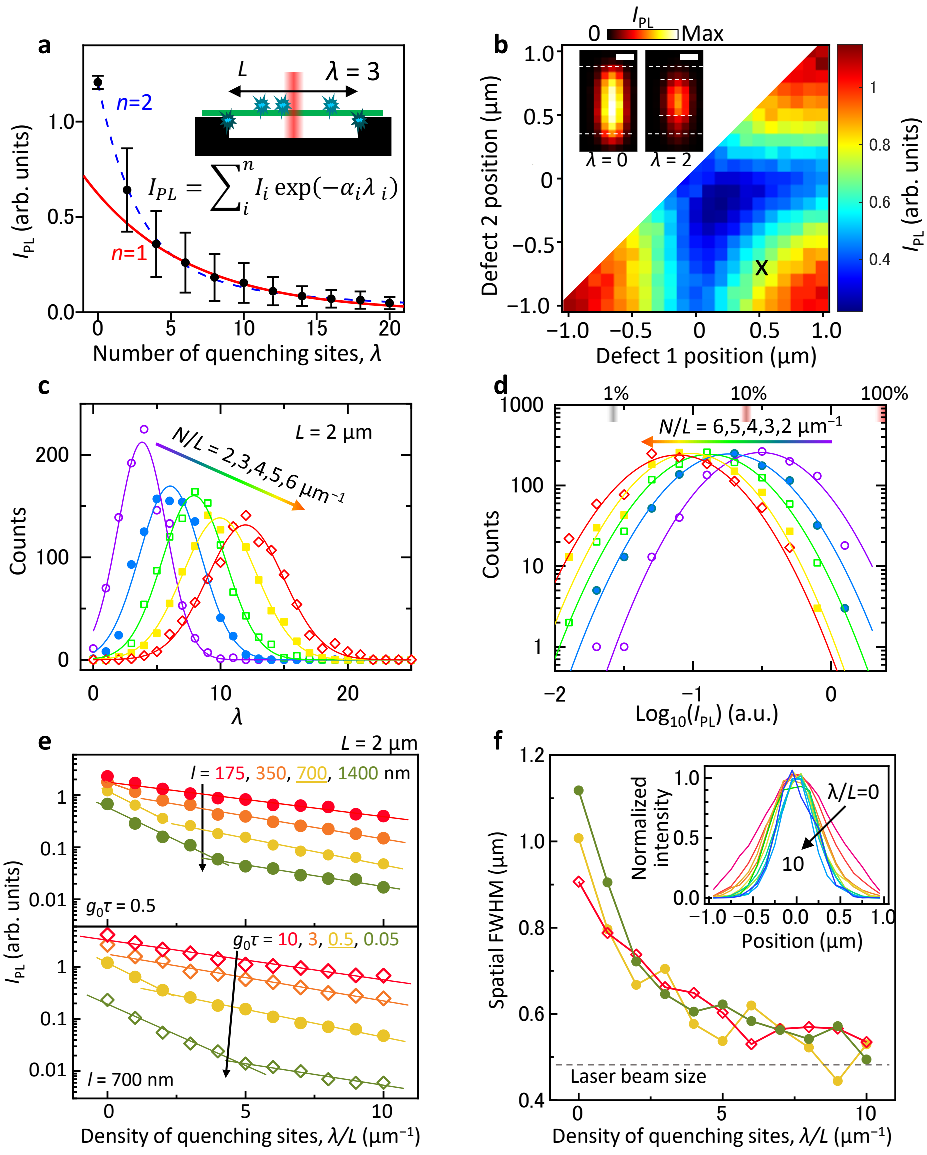

Supplementary Fig. 7 | Monte Carlo simulation on PL intensity that depends on the number of quenching sites. a, Simulated PL intensity as a function of the number of quenching sites λ in a nanotube with a length L of 2 µm. Error bars represent the standard deviation. **b**, Simulated PL intensity as a function of the position of two quenching sites. Inset shows simulated confocal PL images without quenching sites and with two quenching sites at x in the color plot. Scale bars are 500 nm. **c**,**d** Histograms of λ (**c**) and PL intensity (**d**) with different average densities of quenching sites (N/L). Excitation laser beam is fixed at the center of the nanotubes. The percentages at the top represent relative PL quantum yields compared to the intrinsic quantum yield of a sufficiently long CNT without quenching sites. **e**, Dependence of I_{PL} on the density of quenching sites with various exciton diffusion lengths, and with various exciton generation rates $g_0\tau$ (number of generated excitons during the intrinsic lifetime τ). The underscored parameters are used elsewhere. **f**, Simulated spatial FWHM of PL intensity profile along the tube axis (wide field image) *versus* λ/L. Simulation parameters of each plot are the same as in **e**. Inset: Normalized PL intensity profiles with various λ/L.

Supplementary Note 8:

Influence of baking and water rinsing on PL properties

One of the major features of the anthracene-assisted transfer is the absence of high-temperature process and wet process. Anthracene crystals are removed typically at 110ºC, but this temperature can be lowered even to room temperature, though it takes a few days for the full sublimation. Also, the growth and the removal of anthracene crystals are conducted in gas phase, and no wet etching is required to peel the crystals off the substrate. When using polymer films to transfer CNTs [2], spin-coating of the polymer in solvents, etching of the growth substrates, and the removal of polymer by solvents or pyrolysis at high temperature are involved. We have compared the PL intensity of CNTs on PMMA before and after baking at 170ºC, baking at 230ºC, and immersing in distilled water. In all cases, we find that the PL intensity decreased nearly by half (Supplementary Fig. 8). The linewidths also increased with these treatments. These results support the importance of avoiding high-temperature processes and wet processes in the anthracene-assisted transfer.

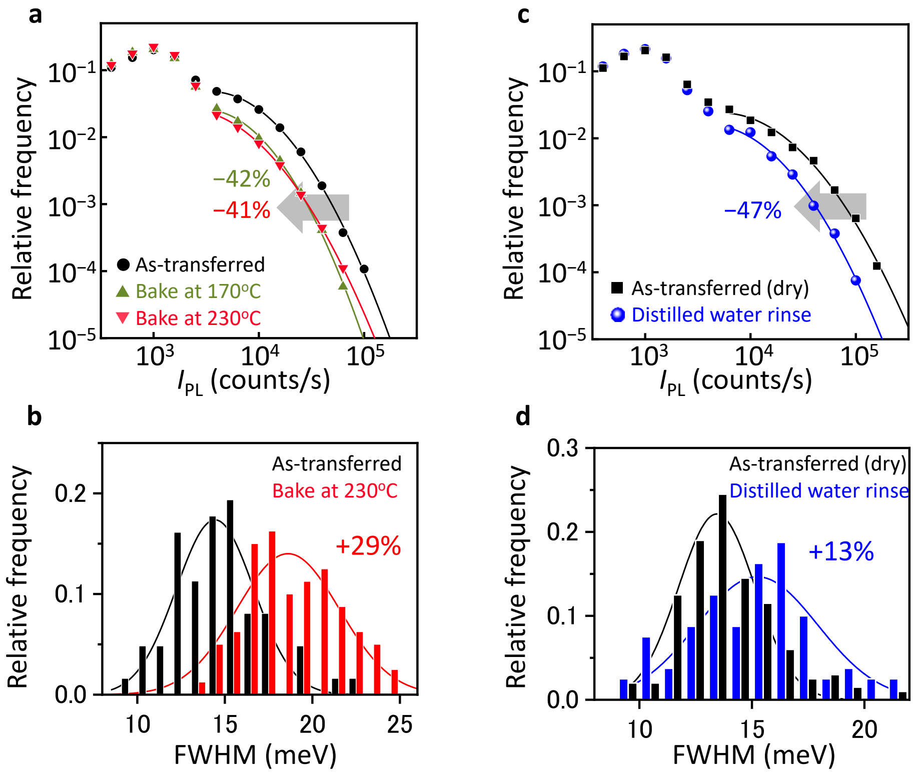

Supplementary Fig. 8 | Effects of baking and water on PL. a, I_{PL} histograms of the CNTs transferred on PMMA. Black circles represent as-transferred CNTs, whereas green and red triangles represent the same sample but after bake at 170 and 230°C for 1 h, respectively. **b**, Spectral FWHM of as-transferred CNTs and those after the bake. **c**,**d**, Similar data set for as-transferred CNTs and the identical CNTs after the immersion in distilled water for 2 h. P = 100 μW.

Supplementary Note 9:

Anthracene-assisted transfer of monolayer WSe_2 over trenches

The advantages of using anthracene as sacrificial layers are also evident in the case of 2D materials, such as h-BN and MoS_2. We first prepared a flake of mechanically exfoliated WSe_2 onto a flat SiO_2/Si substrate (Supplementary Fig. 9a). A half of the WSe_2 flake is monolayer and another half is bilayer. This WSe_2 flake is then picked up on an anthracene crystal. Note that atomically flat surface of the anthracene crystal enhances the adhesion to 2D materials, and thereby enables the picking up of monolayer 2D materials from SiO_2/Si substrates.

We place the WSe_2/anthracene on a Si substrate with trenches, followed by sublimation of the anthracene crystal by mild heating, leaving behind the WSe_2 on the receiving substrate (Supplementary Fig. 9b). A dark-field image in Supplementary Fig. 9c shows that both monolayer and bilayer regions are suspended over the trenches. We have measured PL and Raman spectra from the monolayer (Supplementary Fig. 9d,f) and bilayer (Supplementary Fig. 9e,g) regions, and on the SiO_2/Si surface (green lines) and over the trench (red lines). When comparing the PL intensity of the monolayer part, the suspended region shows significantly higher intensity than on the SiO_2/Si surface, while Raman spectra indicate negligible strain in the suspended region. The steep contrast of the PL intensity and spectral shape between Supplementary Fig. 9d and 9e originates from layer number difference. As the flatness and the sublimation of anthracene crystals facilitates the picking up of 2D materials and prevents surface tension from destroying the 2D materials, respectively, 2D materials transfer can be improved by this technique as much as 1D nanotubes.

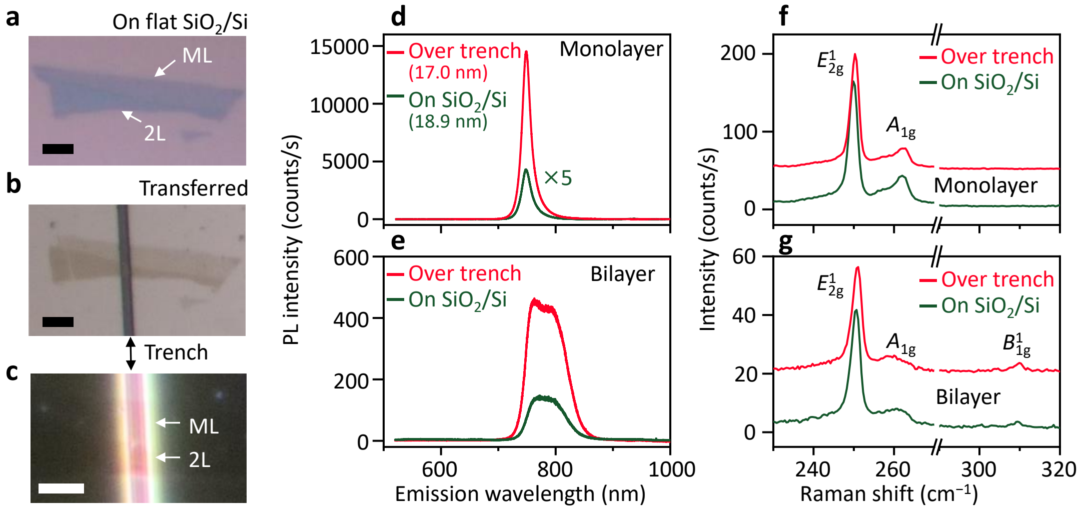

Supplementary Fig. 9 | Anthracene-assisted transfer of monolayer WSe_2. a, Mechanically exfoliated mono- and bilayer WSe_2 on an SiO_2/Si substrate. **b**,**c**, Bright-field (**b**) and dark-field (**c**) optical images of the transferred WSe_2 flake on another SiO_2/Si substrate with a trench. All scale bars are 5 µm. **d**,**e**, PL spectra of the transferred WSe_2. Numbers in parentheses represent FWHM.

The spectrum of the monolayer WSe_2 on an SiO_2/Si substrate is multiplied by 5 times for visual convenience. **f**,**g**, Raman spectra of the transferred WSe_2. Excitation wavelength for PL and Raman spectroscopy is 488 nm, and the excitation powers are 1.5 and 60 µW for PL and Raman spectroscopy, respectively.

Supplementary Note 10:

Air-suspended CNTs prepared by polymer-mediated transfer

Polymer-mediated methods are often used to transfer CNTs onto flat substrates [2]. When there are trenches or slits in receiving substrates, the transferred CNTs tend to break or form bundles with neighboring CNTs due to the surface tension induced by organic solvents for polymer removal or molten polymers at high temperatures. In Supplementary Fig. 10c,d,e, the CNTs are transferred *via* a PMMA film, which has been formed by spin-coating of a 4wt% solution in anisole at 3000 rpm for 30 s. The PMMA film has been removed by annealing in Ar/H_2 atmosphere at 350°C for 6 h. Even when CNTs are still suspended after the polymer removal, the polymer remains on the CNTs, negatively influencing the PL properties (Supplementary Fig. 10f).

Using Raman spectra, we characterized the quality of CNTs transferred *via* PMMA and anthracene. The ratio of D-band intensity to G-band intensity (D/G ratio) in Raman spectra is used as the measure of nanotube quality. Supplementary Fig. 10g–i shows the D/G ratio histograms for as-grown CNTs on a quartz substrate, CNTs transferred with PMMA onto a SiO_2/Si substrate, and CNTs transferred with anthracene onto a SiO_2/Si substrate. As the CNTs transferred *via* anthracene crystals show similar or slightly higher D/G ratios than those *via* PMMA, there is no apparent selectivity for picking up higher quality CNTs, in which a stronger π-π interaction is expected. Note that Supplementary Fig. 10g,h is obtained from the same CNT sample before and after the transfer, and Raman spectra for Supplementary Fig. 10i are measured after SEM observation, which in general increases the D-band intensity of graphitic materials [9].

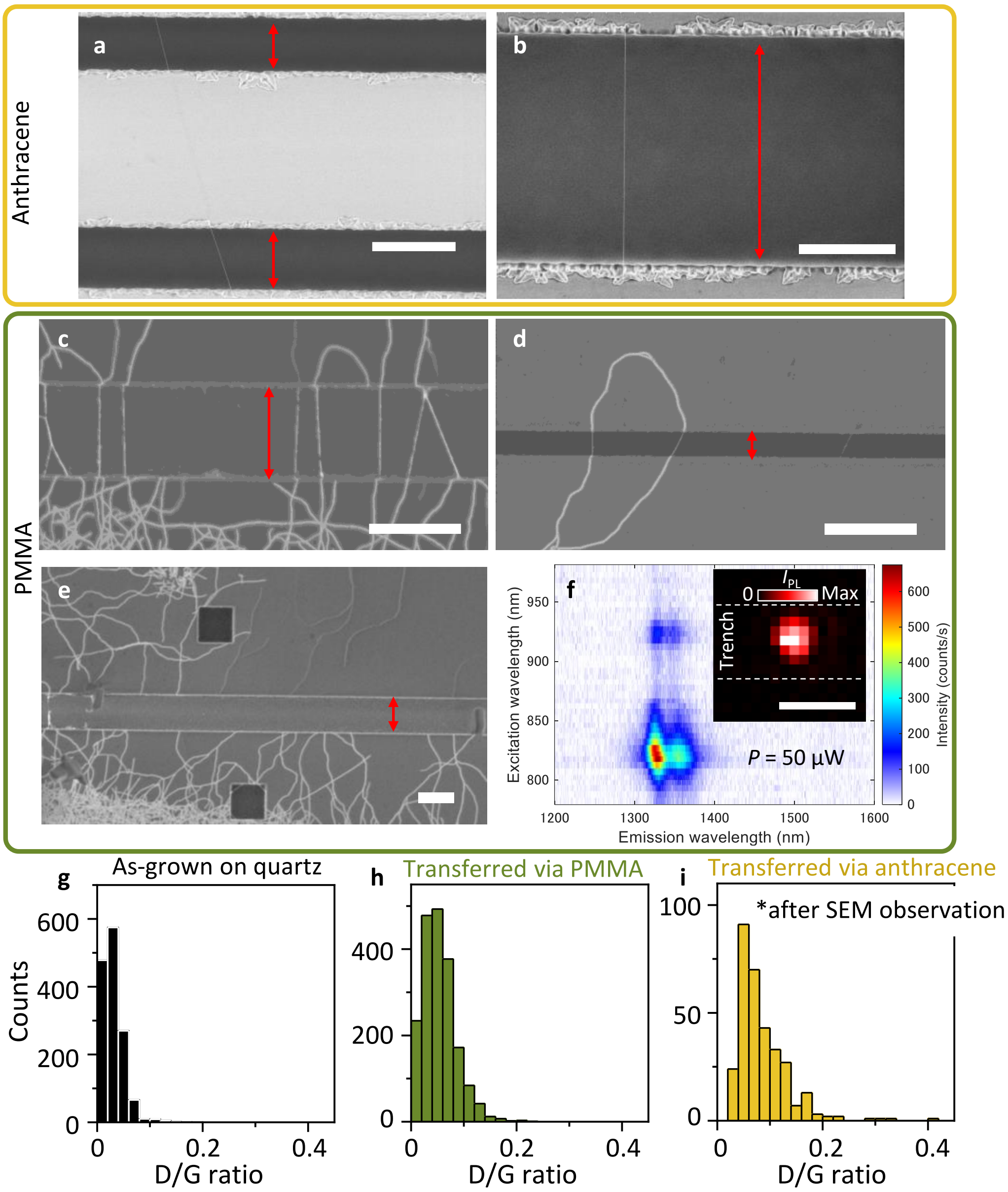

Supplementary Fig. 10 | Comparison of CNTs transferred via anthracene and polymer. a–e, Typical SEM images of air-suspended CNTs that are transferred *via* anthracene crystals (**a**,**b**) or *via* PMMA films (**c**–**e**). Red arrows represent the width of trenches. **f**, Typical PLE map for air-suspended CNTs by the PMMA transfer method. Inset: PL image of the CNT. All the scale bars are 2 µm. **g**–**i**, Histograms of D/G ratios of Raman spectra from as-grown CNTs on a quartz substrate, and those transferred onto SiO_2/Si substrates using PMMA or anthracene sacrificial layers.

Supplementary Note 11:

Repeating deterministic transfer

After the (11,6) nanotube was transferred (Fig. 4b,c), we transferred a (9,7) CNT nearby. In Supplementary Fig. 11b, PL signals at the emission wavelengths λ_{ex} of 1330 and 1433 nm are shown in green and red, respectively, and the laser reflectivity is superimposed in gray scale. Polarization dependence of I_{PL} in the inset of Supplementary Fig. 11b indicates the angle between these tube axes to be ~60 degrees. Supplementary Fig. 11c,d display the PL emission spectra excited at each E_{22} resonance and PLE spectra cut at each E_{11} peak, respectively. These spectra are measured at the same spot, confirming the presence of both (9,7) and (11,6) tubes at the designated position. The (9,7) tube is located even closer to the center of the cross mark than the other tube, indicating the position accuracy in our transfer system.

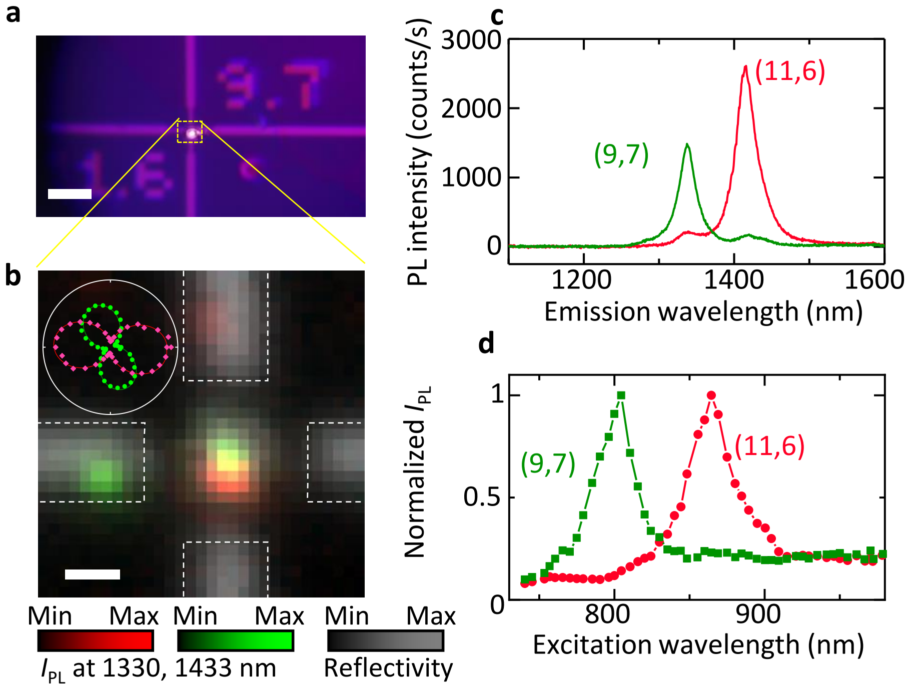

Supplementary Fig. 11 | Multiple transfer of chirality-selected CNTs to a designated position. **a**, Wide-field optical image of a target substrate during the second transfer process. Numbers, such as (9,7) and (11,6), are lithographically patterned metals, and represent the chiral indices of CNTs to be transferred. Bright spot near the center is the laser beam reflection, where PL spectra is obtained in real time for the alignment. Scale bar is 10 μm. **b**, Confocal PL image of transferred CNTs. Intensities at 1330 and 1433 nm, and laser reflectivity are colored in red, green, and gray, respectively. Scale bar is 1 μm. Inset: Polarization dependence of I_{PL}. **c**,**d**, PL emission spectra (**c**) and PL excitation spectra (d) of the transferred CNTs. P = 100 μW and laser polarization is parallel to each CNT.

Supplementary Note 12:

PL properties after the first and the second transfer processes

In Fig. 5, the (10,5) and (13,5) CNTs are transferred in sequence. The characteristics of the (10,5) tube along are shown here in comparison with those after the transfer of the (13,5) tube. When comparing the PL images in Fig. 5b and Supplementary Fig. 12a, there is no obvious difference before and after the transfer of the (13,5) tube. Before the second transfer process, there is no emission at ~1500 nm, as evidenced by Supplementary Fig. 12b,c.

To estimate the exciton transfer efficiency from the (10,5) tube to the (13,5) tube, we first extract the contribution of intertube exciton transfer to the PL intensity by removing the off-resonantly excited component in the (13,5) tube. Comparing the two PL images in Fig. 5a,c, we multiply the PL intensity in Fig. 5a by a coefficient k = 0.294 to match the intensity in Fig. 5c at a position where no exciton transfer is expected (white arrow in Supplementary Fig. 12d). The PL intensity contribution from intertube exciton transfer is then

$$I_{\mathrm{ET}}(x,y) = I_{\mathrm{off},1514}(x,y) - kI_{\mathrm{on},1514}(x,y), \quad (5)$$

where I_{on} (I_{off}) represents the PL intensity at ~1514 nm by on-resonant (off-resonant) excitation at the laser position (x,y). Taking the ratio of the PL intensities in Supplementary Fig. 12d to Fig. 5c, the contribution of the intertube exciton transfer to the (13,5) tube emission is 42% of the total intensity at the blue diamond. Using this ratio (42%) and a PL spectrum taken at the blue diamond in Fig. 5b,c, we can calculate the emission at 1514 nm originating from exciton transfer to be I_{ET} = 1.07×10^4 counts/s, whereas the PL intensity at 1263 nm ($I_{\mathrm{on},1263}$ = 1.27×10^5 counts/s) is the contribution from excitons generated and recombined in the (10,5). By comparing the number of excitons that go through the intertube transfer process and that are initially generated in the (10,5) tube, the exciton transfer efficiency η_{ET} should be calculated as

$$\eta_{\mathrm{ET}} = \frac{\eta^{-1} I_{\mathrm{ET},1514}}{\eta^{-1}(I_{\mathrm{ET},1514} + I_{\mathrm{on},1263})}, \quad (6)$$

where η is the product of the PL collection efficiency and the PL quantum yield for both nanotubes. η_{ET} of 7.8% is obtained from this rough estimation. Note that exciton-exciton annihilation and the difference in the PL quantum yield of each CNT affect the estimation of the exciton transfer efficiency, but these factors are ignored for simplicity.

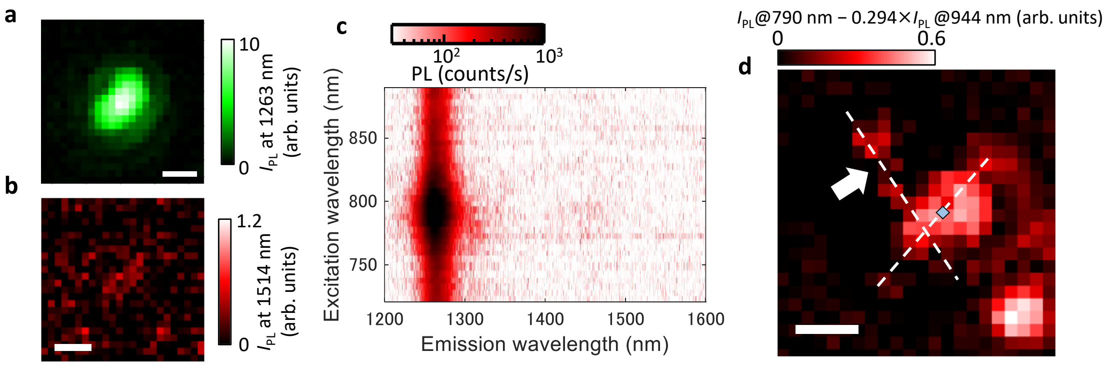

Supplementary Fig. 12 | PL before and after the junction formation. a,**b**, PL image of the (10,5) CNT in Fig. 5 in the main text before the cross junction formation with the spectral windows centered at 1263 nm (**a**) and 1514 nm (**b**). **c**, PLE map for the CNT shown in **a**. **d**, Extracted PL image of the (13,5) tube obtained from Fig. 5a,c, which shows the contribution of the intertube exciton transfer to the PL emission. Scale bars are 1 µm.

Supplementary Note 13:

Periodic strain induced during the CNT growth

In Fig. 5d,e, PL emission from the (13,5) tube is split into two peaks because of strong local strain that has been created during the CNT growth on quartz [8] (see Supplementary Fig. 13a,b). The strain is induced by the difference in thermal expansion coefficients between CNTs and the quartz substrates, and results in the energy shift at E_{ii} resonances and Raman spectra, *e.g.* G-band shifts as shown in Supplementary Fig. 13c. For example, the PL emission peaks can be different by ~100 nm within a single nanotube. Surprisingly, such strain is preserved all the way through the transfer process if the entire length of CNTs remains in contact with the supporting substrates, which is not observed for the polymer-mediated wet transfer technique [2,10]. Difference in G-peak frequency within the transferred nanotube is as large as 12 cm^{-1} (Supplementary Fig. 13d), corresponding to E_{11} energy shift of ~45 meV when the same relation between G-peak position and E_{11} energy (3.72 meV/cm^{-1}) is assumed.

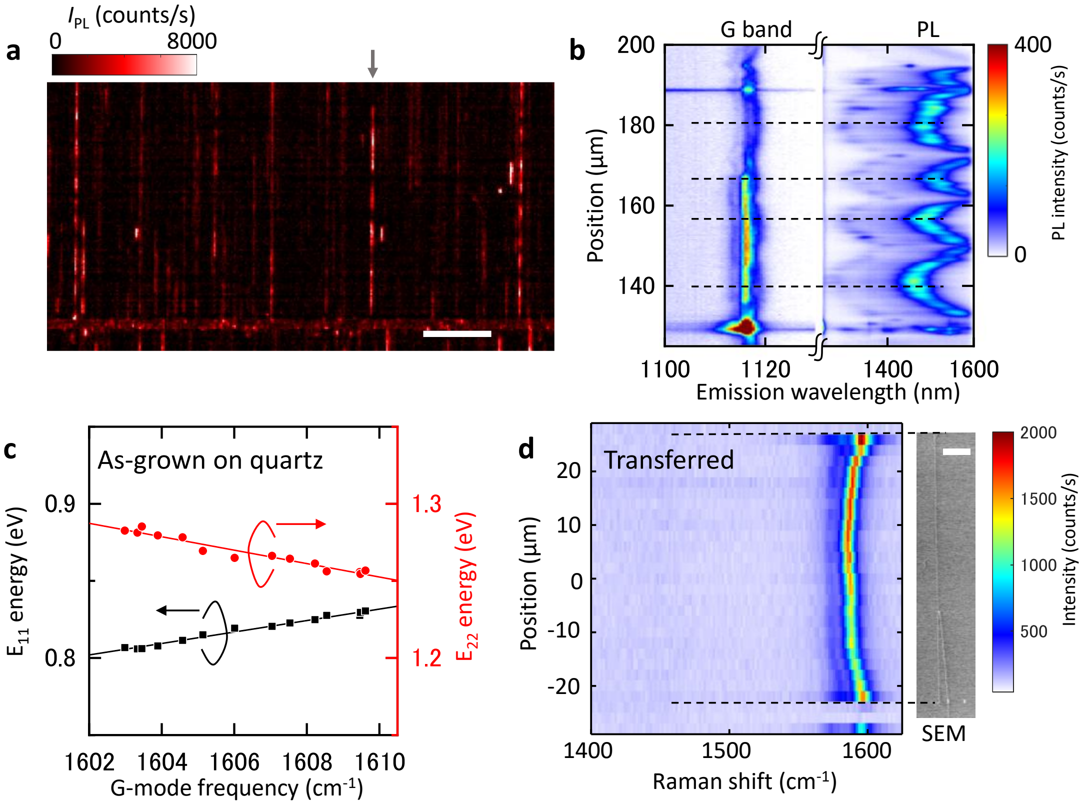

Supplementary Fig. 13 | Strain induced during the CNT growth. a, PL image of as-grown CNTs on quartz substrate. I_{PL} is integrated at 1498 nm with a spectral window of 50 nm. P = 1.2 mW. Scale bar is 20 μm **b**, PL (and Raman) intensity map along the single CNT indicated by the arrow in **a**. Not that there could be multiple CNTs within an excitation laser spot when G-band appears. **c**, E_{11} and E_{22} energies as a function of the peak position of G-band for the CNT shown in **a** and **b**. **d**, Raman intensity map along a transferred CNT from quartz. Scale bar is 5 μm in the inset.

Supplementary References

1. Ye, X. *et al.* Microspacing In-Air Sublimation Growth of Organic Crystals. *Chem. Mater.* **30**, 412–420 (2018).
2. Jiao, L. *et al.* Creation of nanostructures with poly(methyl methacrylate)-mediated nanotransfer printing. *J. Am. Chem. Soc.* **130**, 12612–12613 (2008).
3. Schweiger, M., Zakharko, Y., Gannott, F., Grimm, S. B. & Zaumseil, J. Photoluminescence enhancement of aligned arrays of single-walled carbon nanotubes by polymer transfer. *Nanoscale* **7**, 16715–16720 (2015).
4. Ishii, A., Yoshida, M. & Kato, Y. K. Exciton diffusion, end quenching, and exciton-exciton annihilation in individual air-suspended carbon nanotubes. *Phys. Rev. B* **91**, 125427 (2015).
5. Otsuka, K., Ishii, A. & Kato, Y. K. Super-resolution fluorescence imaging of carbon nanotubes using a nonlinear excitonic process. *Opt. Express* **27**, 17463 (2019).
6. Hertel, T., Himmelein, S., Ackermann, T., Stich, D. & Ḱ, J. C. Diffusion Limited Photoluminescence Quantum Yields in 1-D Semiconductors. *ACS Nano* **4**, 7161–7168 (2010).
7. Harrah, D. M. & Swan, A. K. The Role of Length and Defects on Optical Quantum Efficiency and Exciton Decay Dynamics in Single-Walled Carbon Nanotubes. *ACS Nano* **5**, 647–655 (2011).
8. Ozel, T., Abdula, D., Hwang, E. & Shim, M. Nonuniform Compressive Strain in Horizontally Aligned Single-Walled Carbon Nanotubes Grown on Single Crystal Quartz. *ACS Nano* **3**, 2217–2224 (2009).
9. Teweldebrhan, D., & Balandin, A. A. Modification of graphene properties due to electron-beam irradiation. *Appl. Phys. Lett.* **94**, 013101 (2009).
10. Otsuka, K. *et al.* Digital Isotope Coding to Trace the Growth Process of Individual Single-Walled Carbon Nanotubes. *ACS Nano* **12**, 3994–4001 (2018).
